# Six Mantoux tuberculin skin tests with 1, 2, 5, 10, 20, and 50 units in a healthy male without side-effects – is skin reaction a linear function of tuberculin dose?

**DOI:** 10.1186/1757-1626-1-115

**Published:** 2008-08-20

**Authors:** Ioannis DK Dimoliatis, Christos A Liaskos

**Affiliations:** 1 Department of Hygiene & Epidemiology, Ioannina University Medical School, University campus, 45110, Ioannina, Greece

## Abstract

**Background:**

Tuberculosis remains a serious disease worldwide. Anti-tuberculosis campaigners many times face negative tuberculin skin tests after Bacille Calmette Guérin vaccination. Increasing tuberculin units might be a solution. However, is skin reaction a linear function of tuberculin dose? Are there any side-effects when higher tuberculin doses are administered?

**Case presentation:**

Six simultaneous Mantoux tuberculin skin tests, using 1, 2, 5, 10, 20, and 50 tuberculin units (88 altogether) of purified protein derivative RT23 per 0.1 mL were applied in a healthy male Greek 35-years-old, with known natural *Mycobacterium tuberculosis *primary infection since five years. Skin indurations 72 hours later were 15, 22, 23, 19, 23, and 27 mm respectively.

**Conclusion:**

No linear relation between tuberculin dose and skin reaction observed; skin reaction increased as tuberculin dose increased but with a decreasing rate, especially after 2 TUs, which seem correctly defined for detection of natural infection. No side-effects occurred.

## Background

During eighties the first author (ID), responsible for the antituberculosis campaign in Ioannina prefecture, Greece, faced negative post Bacille Calmette-Guérin (BCG) vaccination tuberculin skin tests (TST) many times, and he had to answer why. Was the BCG vaccine inadequate? Was the inoculation technique inadequate? Was the usual tuberculin dose in TSTs, recommended for detection of the natural infection, also adequate for detection of the artificial BCG-infection?

BCG contains weak bacilli; post-BCG TSTs may need increased tuberculin doses. He increased tuberculin units (TU) [1], supposing that the more the units are the more the reaction is. However, this is not known. In addition, are there any side-effects when higher tuberculin doses are administered?

ID experimented on himself using simultaneously six different dilutions: 1, 2, 5, 10, 20, and 50 TUs per 0.1 mL (88 TUs altogether). None of the three relevant papers [2-4], retrieved (22 May 2008) from Medline using the algorithm (Mantoux OR Imotest OR Tine OR "tuberculin skin test*" OR "tuberculin test*") AND (simultaneous* OR multiple OR "same person" OR "same individual" OR "same patient") AND (dose OR dosage OR unit* OR "TU"), had used more than 15 TUs altogether simultaneously on the same person, compared to 88 TUs in our case.

Tuberculosis remains a serious disease that kills millions worldwide [5-7], especially after human immunodeficiency virus pandemic and worldwide emerging multidrug-resistant strains [6]; moreover it has come back, even where we thought it was eliminated [8]. This self-experiment has lost nothing of its value; contrarily it became evergreen again. Tuberculosis specialists, practising physicians, and immunity researchers might be interested on its results.

## Case presentation

### Methods

**Figure 1:**
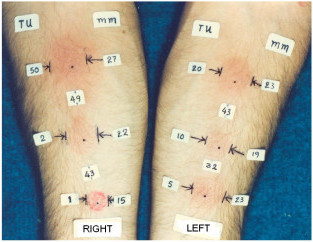
**Six simultaneous tuberculin skin tests on the same person (slightly retouched in 1 TU)**. The hands belong to a healthy Greek male 35, with known natural infection by *Mycobacterium tuberculosis *since 5 years (the first author). The six dots in the middle of erythemas represent the points of intradermal injection (Mantoux technique). The numbers between dots indicate the distance between injection points (in mm). The numbers under the heading 'TU' are the PPD-RT23 tuberculin units per injection (tuberculin dose). The numbers under the heading 'mm' are the induration diameters in mm. The small vertical lines |...| show the induration boundaries, and the arrows towards these lines, transversely to the log axis of the forearm, point to the ballpoint traces made in order to find boundaries with Sokal's method.

In a healthy Greek male physician, 35-years-old, 173 cm height, 72 Kg weight, never-smoker with no more than one or two drinks weekly, with known natural infection since five years, six TSTs were applied in 12 November 1985 (Figure 1). The hands belong to the first author, specialised in Pulmonology at Athens Thorax Diseases Hospital 1976–1980. During this period he had repeatedly negative Mantoux/Sokal TSTs; last negative 10 April 1980. The first positive Mantoux occurred 20 June 1980. Between 11 February 1980 and 11 April 1980 he had no contact at all; thus the most probable period of infection was between 11 and 20 April 1980, his first week in the army, when there was contact with a very probable tuberculosis patient. He received chemoprophylaxis (300 mg isoniazide and 25 mg pyridoxine every morning) from July 1980 to May 1981.

All vials of 1, 2, 5, 10, 20, 50 TU per 0.1 mL purified protein derivative (PPD) RT23 were provided by the Greek Pasteur Institute on the same day, preserved under identical conditions, and administered within a few days after production, long before their expiry date.

The tester was the domiciliary nurse of the Ioannina anti-tuberculosis campaign, with more than twenty years of experience. All six tests were injected lege-artis (one sharp bevelled disposable 25 G needle with a plastic tuberculin syringe per test; intradermal injection of exactly one tenth of a millilitre of PPD tuberculin; 4 mm white blister). Induration was read 72 hours later, using Sokal's technique (Figure 1).

### Results

**Figure 2:**
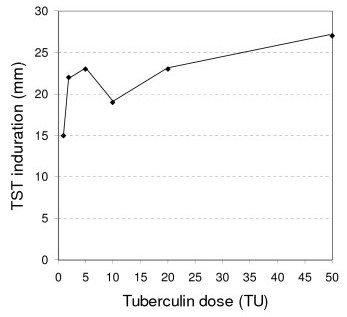
**Tuberculin skin test induration as a function of tuberculin dose**. Six simultaneous Mantoux tests, using 1, 2, 5, 10, 20, and 50 TUs PPD-RT23 per 0.1 mL (see Figure 1).

The results are presented in Figure 1. Skin reaction was not a linear function of tuberculin dose; induration was increased with a decreasing rate as tuberculin dose increases (Figure 2), while an unexpected decrease occurred in the dose of 10 TUs. Erythema (redness) was not measured but it was parallel to induration (Figure 1). No side-effects occurred.

## Discussion

Declining increases have also found Tzimakas et al (1980; four TSTs per person, 1, 2, 5, and 10 TUs PPD per 0.1 mL, in Greek healthy men and tuberculosis patients; the last ones had greater reactions) [9], Tzimakas et al (1984; 30 Greek healthy young women, of which ten were Mantoux tested with 2, ten with 5 and ten with 10 TUs PPD-RT23 per 0.1 mL) [10], Dimoliatis (1987; either 1 or 2 or 10 TUs PPD-RT23 per 0.1 mL Mantoux per group in 279 Greek schoolage children vaccinated with either liquid or lyophilized BCG; 6 groups) [1], Alcaide et al (1992; two Mantoux, 2 and 5 TUs PPD-RT23, in 2575 Spanish individuals) [2], Stuart et al (2000; two TSTs per person, 5 and 10 TUs PPD per 0.1 mL, in 128 Australian health care workers) [3], and Jentoft et al (2001; induration ≥ 15 mm in 32% and 41% of the Mantoux tests with 2 and 5 TUs PPD-RT23 respectively, in Norway) [4]. Tzimakas et al (1980) [9] and Stuart et al (2000) [3] do not clarify the strain of PPD used; perhaps PPD-RT23 and PPD-S respectively, since PPD-RT23 is used in Greece and probably PPD-S in Australia.

The paradox of lower induration at 10 TUs comparing to 5 TUs (Figure 2) may be due to chance, technique errors, insufficient concentration in this particular vial, or unknown factor(s). We do not believe that technique errors occurred (same highly experienced tester, same time, exactly 0.1 mL/test, identical blister per test, identical vial preservation conditions). Chance is highly improbable because the phenomenon was observed again a few days later (November 1985), when 431 children, previously vaccinated with lyophilized BCG (April 1985), were Mantoux tested using the same vials and found the same pattern, in particular a mean diameter in mm 5.8 (standard deviation 4.8) in 74 children tested with 1 TU, 9.3 (4.4) in 15 with 2 TUs, 10.9 (5.7) in 88 with 5 TUs, 10.0 (5.4) in 94 with 10 TUs, 11.4 (5.8) in 78 with 20 TUs, and 14.5 (4.9) in 82 with 50 TUs [11]. Thus the most probable explanation is that something was wrong with this particular 10 TUs batch. Tzimakas et al [9] and Stuart et al [3] did not offer details on reaction sizes and PPD-strain used respectively; thus it is impossible to check whether our decrease in 10 TUs exists also in their data.

The main thought of researcher, when he dared the import of so many TUs in his body (44 times the usual TST dose, and 5 to 6 times the highest bibliography dose), was that much more quantities of (not purified) *mtb *derivatives should exist within the body of the tubercular, and in addition in the lungs and not simply intradermally. The absence of any side effect vindicated this hypothesis. He was also based on the already use of denser dilutions [1], where he concluded that the TST reaction is not a linear function of the tuberculin dose, but rather a decreasingly increasing relation, compatible with the known in Immunology antigen-antibody curve [6]. This hypothesis was also confirmed with this self-experiment: the skin reaction is an increasing function of the tuberculin dose but with a decreasing rate, especially after 2 TUs. It is worth noting that because of this decreasing rate the recommended dose for routine detection of the natural infection has correctly been defined to be 2 TUs PPD-RT23 per 0.1 mL: while the gain in mm of induration is big until this dose, it is negligible beyond this.

This is a case report. However, these results were repeated in six groups of children tested with the six vials used in Figure 1[11], and are in accordance to other research [1-4,9,10].

## Conclusion

After six simultaneous Mantoux tuberculin skin tests with 1, 2, 5, 10, 20, and 50 TUs in a healthy male 35, i.e. after simultaneous injection on the same person 44 times more TUs than the usual TST dose of 2 TUs PPD-RT23, (i) no side-effects occurred; (ii) no linear relation between tuberculin dose and skin reaction observed; the later increased as tuberculin dose increased but with a decreasing rate, especially after 2 TUs, which (iii) seem correctly defined for detection of natural infection.

## Abbreviations

BCG: Bacille Calmette Guérin; mL: Millilitre; *mtb:Mycobacterium tuberculosis*; PPD: Purified protein derivative; RT23: the name of the tuberculin used; TST: Tuberculin skin test; TU: Tuberculin unit.

## Consent

"Written informed consent was obtained from the patient for publication of this case report and accompanying images. A copy of the written consent is available for review by the Editor-in-Chief of this journal."

## Competing interests

The authors declare that they have no competing interests.

## Authors' contributions

ID conceived the idea and performed the experiment. CL performed the medline search and wrote the first draft. All authors read and approved the final manuscript. ID is the guarantor of the paper.
